# Accounting for the valley of recovery during post-stroke rehabilitation training *via* a model-based analysis of macaque manual dexterity

**DOI:** 10.3389/fresc.2022.1042912

**Published:** 2022-12-20

**Authors:** Jun Izawa, Noriyuki Higo, Yumi Murata

**Affiliations:** ^1^ Faculty of Engineering, Information and Systems, University of Tsukuba, Tsukuba, Japan; ^2^ Neurorehabilitation Research Group, Human Informatics and Interaction Research Institute, National Institute of Advanced Industrial Science and Technology (AIST), Tsukuba, Japan

**Keywords:** computational neurorehabilitation, state-space model, generalization, spontaneous recovery, use-dependent recovery

## Abstract

**Background:**

True recovery, in which a stroke patient regains the same precise motor skills observed in prestroke conditions, is the fundamental goal of rehabilitation training. However, a transient drop in task performance during rehabilitation training after stroke, observed in human clinical outcome as well as in both macaque and squirrel monkey retrieval data, might prevent smooth transitions during recovery. This drop, i.e., recovery valley, often occurs during the transition from compensatory skill to precision skill. Here, we sought computational mechanisms behind such transitions and recovery. Analogous to motor skill learning, we considered that the motor recovery process is composed of spontaneous recovery and training-induced recovery. Specifically, we hypothesized that the interaction of these multiple skill update processes might determine profiles of the recovery valley.

**Methods:**

A computational model of motor recovery was developed based on a state-space model of motor learning that incorporates a retention factor and interaction terms for training-induced recovery and spontaneous recovery. The model was fit to previously reported macaque motor recovery data where the monkey practiced precision grip skills after a lesion in the sensorimotor area in the cortex. Multiple computational models and the effects of each parameter were examined by model comparisons based on information criteria and sensitivity analyses of each parameter.

**Result:**

Both training-induced and spontaneous recoveries were necessary to explain the behavioral data. Since these two factors contributed following logarithmic function, the training-induced recovery were effective only after spontaneous biological recovery had developed. In the training-induced recovery component, the practice of the compensation also contributed to recovery of the precision grip skill as if there is a significant generalization effect of learning between these two skills. In addition, a retention factor was critical to explain the recovery profiles.

**Conclusions:**

We found that spontaneous recovery, training-induced recovery, retention factors, and interaction terms are crucial to explain recovery and recovery valley profiles. This simulation-based examination of the model parameters provides suggestions for effective rehabilitation methods to prevent the recovery valley, such as plasticity-promoting medications, brain stimulation, and robotic rehabilitation technologies.

## 1. Background

Motor training for impaired motor skills is a fundamental component of rehabilitation therapy. However, the mechanisms behind how motor skills are improved with rehabilitation training remain ambiguous. One critical issue for a mechanistic understanding is the distinction between true recovery and compensation. True recovery refers to recovery that achieves the same motor pattern as the prestroke movements with the recruitment and reorganization of undamaged perilesional regions, whereas compensation refers to an alternative movement pattern (1) to accomplish a given motor task, which is often categorized as a different type of motor skill (2).

For instance, in Murata 2008 (3), after macaques received damage in the primary motor cortex that disrupted digit representation, when performing a task of retrieving food rewards with the thumb and index fingers, the monkeys initially exhibited a compensation strategy where only the index finger was extended and flexed while keeping the thumb finger flexed; subsequently, true recovery of the precision grip using flexion of both the index and thumb fingers appeared. Notably, in the course of the transition from using the compensatory grip to using the precision grip, the monkeys' performance exhibited a significant drop in the food retrieval success rate (4), as if there is a “valley of recovery” existing between compensation and true recovery. This puzzling phenomenon, observed also in squirrel monkey rehabilitation training (5, 6), has been recognized in clinical trials, where patients were encouraged to use more effortful original motor skills and reducing the use of easy compensatory skills becomes a central issue (7). When the presented skill was switched, the patients often exhibited a drop in the success rate (7, 8). Why this valley of recovery occurs and how the brain moves beyond this valley of recovery are unclear.

After stroke, enhanced long-term potentiation (LTP) and homeoplastic mechanisms in the perilesional region lead to remodeling of the motor map (9–11). In addition to functional reorganization in the peri-infarct motor cortex, training-induced (i.e., use-dependent) plastic changes occur in remote, intact, cortical areas (12) such as the premotor cortex (13, 14) and motor areas in the intact hemisphere (10, 15). Notably, training-induced plastic changes in the motor representation in both the perilesional region and the other cortical areas are also caused by the repetition of compensatory motor skills (16, 17). These simultaneous plastic changes underlying true motor recovery and compensation suggest that true recovery and compensation might compete for the limited neural resources in the cortex post stroke (18). If such competition exists, recovery should be taken over by one initially selected motor skill, which should prevent escaping the recovery valley. However, in Murata et al. (3), the recovery valley was eventually overcome, and there was a successful transition between the two skills. Thus, one possible hypothesis regarding the mechanisms supporting the overcoming of the recovery valley is that training with a compensatory skill may generalize to the acquisition of precision skills in the same way that learning generalization across different motor skills (19) rather than interference (18), occurs.

To test this hypotheses, we adopt a computational modeling approach that has been used in studies of motor skill learning (20). In this framework, the formed motor skill is represented by state variables (11), and motor skill learning is modeled as the update of these states in each trial, by which the properties of motor skill learning of the human participants were quantitatively examined after estimating learning parameters from the measured behavioral data (21, 22). In this framework, our hypothesis of the generalization factor between two motor skills was implemented as an interaction term between two skills. This implementation is reasonable if we consider two overlapping motor primitives for two skills (1, 19). Since a population coding with cosin tuning functions for fingers was found in the primary motor cortex where the activation of the thumb partially activated the index finger (23), and since the compensatory grip uses mainly the index finger and the precision grip uses both the index and the thumb finger, the motor primitives of the precision grip is partially shared with that of the compensatory grip. Thus, this approach elucidated interactions in the update processes between two motor skills (19) and the spontaneous recovery of the skill update without the experience of task errors (24). Thus, we considered that this approach provides a mechanistic understanding of the interplay among the development of precision skills, that of compensatory skills, and spontaneous recovery. To determine crucial factors for neurorehabilitation of motor skills, we fit different types of state-space models to previously published monkey motor recovery data that exhibited this overcoming the recovery valley (3) and identified the latent structures and the interplay among factors contributing to the recovery process. Note that the scope of our modeling in this paper is to elucidate the process of how the two motor skills recover with interacting each other while the choice of the two skills were changed during the recovery assuming that the decision-making policy was updated in that way. Thus, we did not model the decision-making process and used the measured skill selection probability as an input to the model of skill recovery.

## 2. Materials and methods

### 2.1. Behavioral experiments and data

Previously published data from the last author and colleagues (3) with monkeys that received lesions in the digit-related areas in the motor cortex were reanalyzed for this study. All experimental protocols were approved by the National Institute of Advanced Industrial Science and Technology Animal Care and Use Committee, as stated in the previously published paper (3). After the preferred hand was identified and received enough training (longer than 10 days) for the precision grip task, the monkeys received lesion in the hand areas in the primary motor cortex. Then, the monkeys were trained with a Klüver board to retrieve food pellets from a well over consecutive days. In the training sessions, the well size was fixed for a particular day, and it was progressively changed from the largest well to the smallest well across days; when the cumulative number of pellets retrieved from a given well size exceeded 1,000, the well size was changed to the next smaller size the following day. Meanwhile, two sets of test sessions were given each day, where 25 pellets were placed pseudo-randomly into the well, five in each of the five wells of different sizes. These test and training sets were delivered 5 days per week, starting 6–10 days after the lesion. Numbers of training trials and probabilities of successful food taking for each well size during the test were counted and calculated manually.

The video footage of the grip skill performance in these test sessions was visually investigated frame-by-frame, and behavior in each trial was manually categorized as either precision grip or compensatory grip. A precision grip was defined as a grip movement that exhibited flexion and extension of all the thumb and index finger joints to pinch the pellet by the tips of these fingers (i.e., terminal opposition). A compensatory grip was defined as a grip movement that predominantly used the index finger extension and flexion, kept the thumb joint unused and pinched the pellet with the tip of the index finger and the dorsum of the thumb. These two grips were classified based on frame-by-frame visual inspection of recorded video images. We had the recorded video image data for three trained monkeys reported in (3), but only two out of three monkeys' data (Monkey-R and Monkey-N) were clear enough to visually classify these two grips: The camera position was too far away in one monkey. Here, we reanalyzed these two (Monkey-R and Monkey-N) monkeys' selection probability of the two skills and the success probability of pellet retrieval for each well size. These two monkeys' lesions were around the hand digit areas, but the two lesion sizes were slightly different: That of Monkey-N was broader than that of Monkey-R, covering wrist and forearm areas. Thus, the recovery profiles of these two monkeys were slightly different (Figures 2,4).

Monkey-R received postlesion training over 45 days. Ten days of the test sessions were carefully selected to capture the transition between the two skills and the recovery valley. To this end, many days were selected from the period that exhibited the valley, whereas a small number of days were selected from the period that exhibited the stable reward probability. The selected days of Monkey-R were days 12, 16, 21, 24, 31, 33, 34, 35, 38, and 44. Monkey-N received training over 60 days. The ten days of test sessions that were selected were days 10, 20, 32, 37, 38, 39, 40, 41, 53, and 61.

For the model examination described below, we used action selection probabilities and success counts for each well of selected days that were analyzed from video recordings. In addition, for all training and test sessions that did not have video records, we used the total trial number of each training session, and the success counts for each well of those sessions. We assumed that the action selection probability was constant between the selected days.

### 2.2. Computational models

#### 2.2.1. General framework and the model of success probability

**Figure 1:**
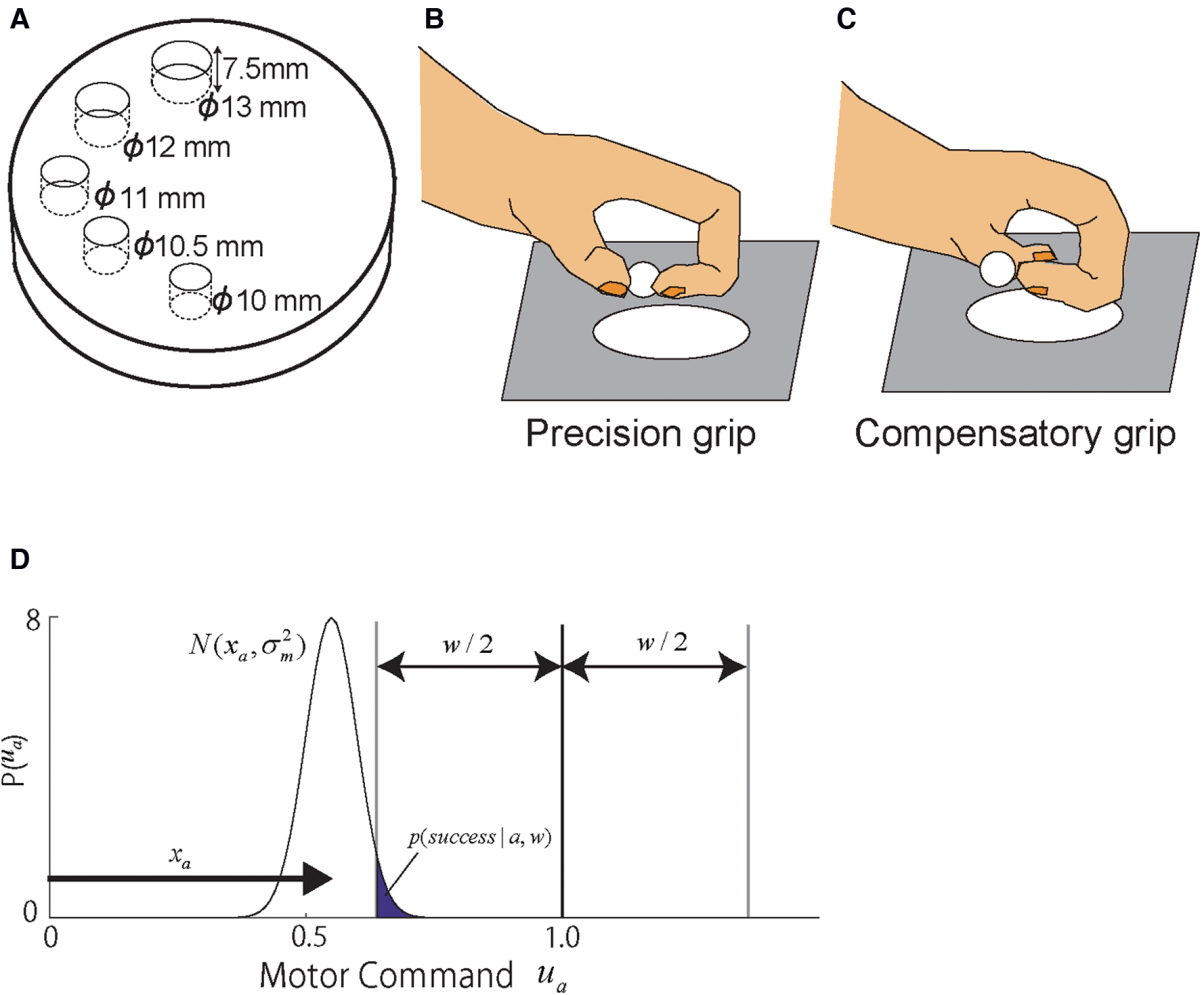
Overview of the task. (**A–C**) Schematic drawings of the Klüver board (**A**) and monkey hand postures during grasping (**B,C**). (**A**) The Klüver board containing cylindrical wells of five different diameters was used in both training and test sessions. (**B,C**) The monkeys used two different grip strategies, precision (**B**) and compensatory grips (**C**), during recovery from motor cortex damage. Reproduced from Figures 1, 8 of Murata et al., 2008. (**D**) Motor command space and reward function defined for the modeling. The skill motor command $u_{a}$ of each skill's space a={p,c} was spanned between 0 and 1 which was normalized by each skill's ideal motor commands such that zero achievements of skill are denoted by 0, and the ideal achievement is denoted by 1. The motor command is generated colored by the motor noise, $u_{a}∼N(\mu_{a},\sigma_{a}^{2})$, where $\mu_{a}$ is the performance level of the skill and $\sigma_{a}^{2}$ is the variance of the skill. The reward area was set at the ideal motor command 1.0 with the well-size ±w/2. The success probability was counted by how many times the stochastic motor commands reach the reward region, which was the shaded area of the motor command distribution: p(success|a,w). *Copyright at JNP https://journals.physiology.org/author-info.permissions*.

In the framework of the state-space model developed to analyze motor learning processes in neurotypical individuals (21) and in individuals with chronic stroke (25), as well as motor skill learning in individuals with chronic stroke (26), the motor memory of skill is presented by the state variable (e.g., x). To apply this framework to analyze the retrieval profile, we hypothesized that the development of the representation of each skill updated by the training is represented by the state variable: $x_{p}$ for the precision grip and $x_{c}$ for the compensatory grip. These state variables take zero at the start of the postlesional training and increase over days as the two skills develop. This formed skill representation is latent to the experimenter but can be estimated through the motor skill executed in the test sessions denoted by $\left[u_{p},u_{c}\right]$. We assumed that the executed motor commands $\left[u_{p},u_{c}\right]$ are generated by the skill performance $\left[\mu_{p},\mu_{c}\right]$ which are generated by the training-induced skill memory in the perilesional cortical region $\left[x_{p},x_{c}\right]$ corrupted by noise (27): $u_{\left[p,c\right]}∼N(\mu_{\left[p,c\right]},\sigma_{\left[p,c\right]}^{2}),\mu_{\left[p,c\right]}=x_{\left[p,c\right]}$, where $\sigma_{\left[p,c\right]}$ are the standard deviations of the motor execution noise that characterize the different accuracies of grip skills between the precision and the compensatory grips. A somewhat redundant notation for the skill level $\mu_{\left[p,c\right]}$ and the internal representation $x_{\left[p,c\right]}$ is to extend this model for more complex model described in the below section. We formulated the skill execution problem as the problem to bring the motor command $\left[u_{p},u_{c}\right]$ to a certain target value. Here, the center of the target for the skill movement is set to 1 for simplicity. Our interest is processes of formation of skill representations $\left(x_{p},x_{c}\right)$. The success probability is proportional to how likely the executed skill hit the target centered at 1 with the width *w* scaled by the motor execution noise $(\mathrm{SD}=\sigma_{\left[p,c\right]})$, which is modeled as the cumulative probability of the normal distribution that enters the goal area (i.e., exceeding 1−w/2, erf function shown in Figure 1):

(1) $$p(\mathrm{success}|a,w)=\left(1-\frac{1}{\sigma_{a}\sqrt{\left(2\pi \right)}}\int_{-\infty }^{1-w/2}e^{\left(-{\left(t-\mu_{a}\right)}^{2})/(2\sigma_{a}^{2}\right)}dt\right),$$

where *a* takes either *p* or *c* depending on the monkey's action selection between the precision and the compensatory grips. The well size *w* takes a value proportional to the given well size in the experiment (13, 12, 11, 10.5, and 10 mm) scaled by a parameter $w_{0}$ such that $w=w_{0}\cdot [13,12,11,10.5,10]$ mm. Since, according to the Equation 1 and Figure 1D, the task performance is determined by a balance between the task demand $w_{0}$ and the size of the motor noise $\sigma_{\left[p,c\right]}$, we supposed that this task demand $w_{0}$ is constant for two grip skills and set only $\sigma_{\left[p,c\right]}$ as free parameters. By checking goodness of fit (likelihood) described below, $w_{0}$ was adjusted manually and in the end, $w_{0}=0.001$. This manual search of a reasonable task demand scale helped reduce the number of free parameters.

#### 2.2.2. The model for training-induced recovery without spontaneous recovery (training only)

Training-induced plasticity and corresponding increases in motor skill level $\left(\mu_{p},\mu_{c}\right)$ are modeled as the update of the state variables $\left(x_{p},x_{c}\right)$ by a single trial experience of using each skill on a training trial *k*. In this model, the motor commands $u_{p},u_{c}$ are the function of the motor skill level $\mu_{p},\mu_{c}$ which are generated by the training-induced update of state variable $x_{p},x_{c}$:

(2) $$\begin{array}{c} x_{p}^{\left(k+1\right)}=\alpha_{p}x_{p}^{\left(k\right)}+\beta_{p}{\mathrm{use}}_{p}^{\left(k\right)}+\beta_{\mathrm{int}}{\mathrm{use}}_{c}^{\left(k\right)},\;\\ x_{c}^{\left(k+1\right)}=\alpha_{c}x_{c}^{\left(k\right)}+\beta_{\mathrm{int}}{\mathrm{use}}_{p}^{\left(k\right)}+\beta_{c}{\mathrm{use}}_{c}^{\left(k\right)},\; \end{array}\;$$

(3) $$\begin{array}{c} \mu_{p}=x_{p},\mu_{c}=x_{c},\;\\ u_{p}∼N(\mu_{p},\sigma_{p}^{2}),\;\\ u_{c}∼N(\mu_{c},\sigma_{c}^{2}),\; \end{array}\;$$

where $\left[\alpha_{p},\alpha_{c}]\;(\alpha_{\left[p,c\right]}\leq 1\right)$ represents the retention (forgetting) rate between two trials and $\left[\beta_{p},\beta_{c}](0\leq \beta_{\left[p,c\right]}\leq 1\right)$ represents the update rate for the single experience of the training with the use of each skill (28, 29). Here, we count each trial with $\mathrm{us}e_{a}=1$ when the monkey performs either precision or the compensative grip. Thus, a single trial experience of the skill increases the skill level, while the absence of experience decreases it. The estimated interaction term of the training effects between two skills is denoted by $\beta_{int}$.

On days when monkeys did not receive any training, memories were not updated, but decayed following: $x_{p}^{\left(\mathrm{day}+1\right)}=\alpha_{p}x_{p}^{\left(\mathrm{day}\right)},x_{c}^{\left(\mathrm{day}+1\right)}=\alpha_{c}x_{c}^{\left(\mathrm{day}\right)}$.

The parameters $\theta =[\alpha_{p},\alpha_{c},\beta_{p},\beta_{c},\beta_{\mathrm{int}},\sigma_{p},\sigma_{c}]$ were estimated to maximize the likelihood of the data measured in the test sessions regarding the success and failure of the selected grasping skill movements. This estimation was conducted independently for each monkey.

#### 2.2.3. Model for spontaneous-recovery enhanced training-induced recovery

The alternative mechanism of motor recovery is spontaneous recovery by which the skill $\mu_{a}(a=\{p,c\})$ develops across days after the lesion (day) independent of training and use. In this model, the training-induced memory update equation is the same as that of the training only model (Equation 2). The contribution of day dependent effect is plugged into the motor output equation (Equation 3). The spontaneous recovery caused by endogenous plasticity is likely to be nonlinear as a function of the postlesional periods (30). Nonlinearly, this might follow an inverse exponential-like function (31), which is still unclear. To account for this nonlinearity and to examine the type of nonlinearity, the model is categorized by six sub-models, depending on how this factor is implemented.

Multiplicative logarithmic function [*log(day)]:

(6) $$\begin{array}{c} sp=\log(\mathrm{day}),\;\\ \mu_{\left[p,c\right]}=x_{\left[p,c\right]}\cdot k_{sp}\frac{sp}{\max(sp)},\;\\ u_{\left[p,c\right]}∼N(\mu_{\left[p,c\right]},\sigma_{\left[p,c\right]}^{2}).\; \end{array}$$

Additive logarithmic function [+log(day)]:

(7) $$\begin{array}{c} sp=\log(\mathrm{day}),\;\\ \mu_{\left[p,c\right]}=x_{\left[p,c\right]}+k_{sp}\frac{sp}{\max(sp)},\;\\ u_{\left[p,c\right]}∼N(\mu_{\left[p,c\right]},\sigma_{\left[p,c\right]}^{2}).\; \end{array}$$

Multiplicative linear function(*day):

(8) $$\begin{array}{c} sp=\mathrm{day},\;\\ \mu_{\left[p,c\right]}=x_{\left[p,c\right]}\cdot k_{sp}\frac{sp}{\max(sp)},\;\\ u_{\left[p,c\right]}∼N(\mu_{\left[p,c\right]},\sigma_{\left[p,c\right]}^{2}).\; \end{array}$$

Additive linear function (+day):

(9) $$\begin{array}{c} sp=\mathrm{day},\;\\ \mu_{\left[p,c\right]}=x_{\left[p,c\right]}+k_{sp}\frac{sp}{\max(sp)},\;\\ u_{\left[p,c\right]}∼N(\mu_{\left[p,c\right]},\sigma_{\left[p,c\right]}^{2}).\; \end{array}$$

Multiplicative exponential function (*exp(day)):

(10) $$\begin{array}{c} sp=\exp(\mathrm{day}),\;\\ \mu_{\left[p,c\right]}=x_{\left[p,c\right]}\cdot k_{sp}\frac{sp}{\max(sp)},\;\\ u_{\left[p,c\right]}∼N(\mu_{\left[p,c\right]},\sigma_{\left[p,c\right]}^{2}).\; \end{array}$$

Additive exponential function (+exp(day)):

(11) $$\begin{array}{c} sp=\exp(\mathrm{day}),\;\\ \mu_{\left[p,c\right]}=x_{\left[p,c\right]}+k_{sp}\frac{sp}{\max(sp)},\;\\ u_{\left[p,c\right]}∼N(\mu_{\left[p,c\right]},\sigma_{\left[p,c\right]}^{2}).\; \end{array}$$

Therefore, for these models accounting for spontaneous recovery effects, parameters to estimate are $\theta =[\alpha_{p},\alpha_{c},\beta_{p},$ $\beta_{c},\beta_{int},\sigma_{p},\sigma_{c},k_{sp}]$.

### 2.3. Parameter estimations and comparison of models

The parameters were estimated by the maximum likelihood method. A simulation of each model was run using the actual sequence of well sizes presented to each monkey during the training sessions. Each simulation run generated the sequence of the memory $x_{a}$, skill level $\mu_{a}$ and p(success|a,w) across the postlesional training period. This sequence of $p_{a,w}^{\mathrm{day}}=p(\mathrm{success}|a,w)$ over the training days predicts the probability of each action's success for each well size. From $p_{a,w}^{\mathrm{day}}$ and the action selection probability for the precision grip p(p) and for the compensation grip p(c), we can compute the marginalized success probability over two actions: $p_{w}^{\mathrm{day}}=p(p)p_{\;p,w}^{\mathrm{day}}+p(c)p_{c,w}^{\mathrm{day}}$.

For all test days, we had success counts for each well size $s_{w}^{\mathrm{day}}$ out of the ten test trials for each well. Thus, the log-likelihood function of the binomial distribution (32) with the marginalized success probability $p_{w}^{\mathrm{day}}$ for each well-size and each day, when a model parameter is θ, is represented as follows:

$$l^{NV}(\theta {)}_{w}^{\mathrm{day}}=\log[C(10,s){\left(p_{w}^{\mathrm{day}}\right)}^{s}{\left(1-p_{w}^{\mathrm{day}}\right)}^{\left(10-s\right)}],$$

where C(n,s) denotes the number of *s*-combinations from a given set of 10 elements and NV stands for “no-video”, described below.

For critical days exhibiting recovery valleys, we had the analyzed data of video footage composed of the selection count $n_{a,w}^{\mathrm{day}}$ and the success count for each action, each well, and each day. The log likelihood function of the binomial distribution with success probability $p_{a,w}^{\mathrm{day}}$ is

$$l^{V}(\theta {)}_{a,w}^{\mathrm{day}}=\log[C(n,s){\left(p_{a,w}^{\mathrm{day}}\right)}^{s}{\left(1-p_{a,w}^{\mathrm{day}}\right)}^{\left(n-s\right)}],$$

where *V* indicates the data from the video footage.

Parameter set θ was estimated to maximize the sum of the log-likelihood over actions, well sizes, and analyzed test days: $L(\theta )=\sum_{\mathrm{day}\in NV}^{}\sum_{w}\sum_{a}l^{NV}(\theta {)}_{w}^{\mathrm{day}}+\sum_{\mathrm{day}\in V}^{}\sum_{w}\sum_{a}l^{V}(\theta {)}_{w}^{\mathrm{day}}$.

To find the best fit θ, we used the nonlinear optimization routine of matlab (fmincon). The 95% confidence interval of the estimated parameter was approximated by $\mathrm{CI}=2\sqrt{diag\{H^{-1}\}}$ where *H* is the Hessian matrix of the log-likelihood evaluation at the maximum.

To compare the predictability of the models, the Akaike information criterion (AIC) was computed by the formula AIC=2k−2L(θ), where *k* is the number of estimated parameters (size of θ).

## 3. Results

### 3.1. Monkey-R

#### 3.1.1. Behavioral data

**Figure 2:**
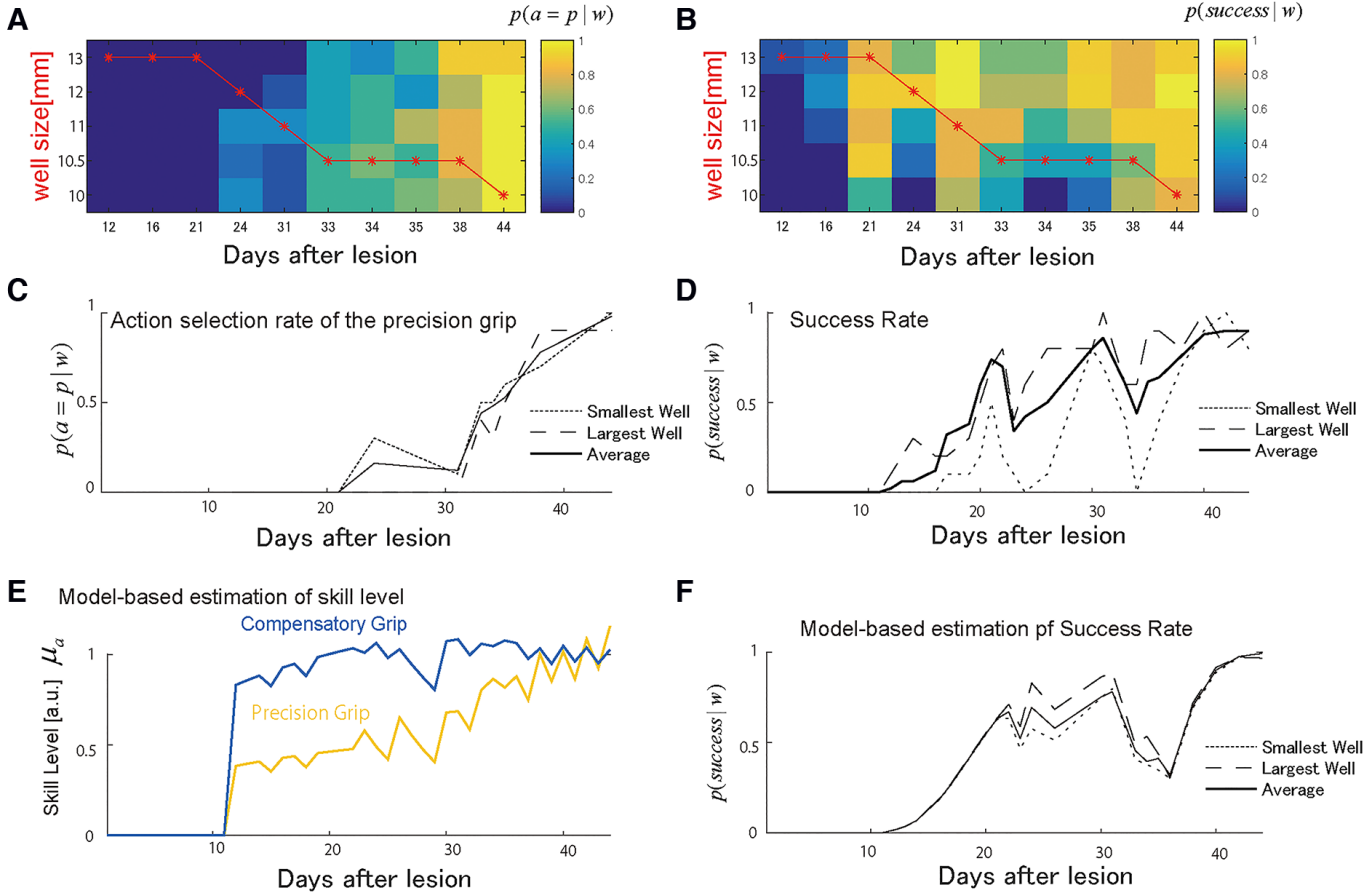
Choice of grasping behaviors for monkey-R. (**A**) Probability of selecting the precision grip in the test sessions over days for each well size. This probability was used to compute the estimated total number of uses each day between test sessions. The red line indicates the presented well size in the training sessions. (**B**) The probability of success in the test sessions over days for each well size. (**C**) The average of the action selection probability of precision grip (i.e., action policy) was plotted for the smallest and the largest well. (**D**) The average success rate was plotted for the smallest and the largest well. (**E**) The estimated development of skill level $\mu_{c},\mu_{p}$ from the selected computational model. The yellow line denotes the precision grip, and the blue line denotes the compensatory grip. (**F**) The predicted success rate for the largest well (the long dashed), the smallest well (the short dashed), and average sized well (solid). *Copyright at JNP https://journals.physiology.org/author-info.permissions*.

Monkey-R (weight: 5.8 kg, dominant hand: left) received a lesion disrupting the grasping representation in M1 of the right hemisphere by injection of ibotenic acid after receiving prelesion training for 13 days. The probability of selecting either the precision grip or compensatory grip in the test session changed over the training sessions such that the monkey initially selected the compensatory grip and switched to the precision skill after the day 30 (Figures 2A,C). The well size had less influence on this skill selection in the test sessions, as shown in the overlapped plots in the probability of action selection among the largest well, the smallest well, and the average of all well sizes shown in Figure 2C. The skill switching started around day 20 when the well size in the training session (shown by the red line) started to change with the lowest demand well (larger on the vertical axis of Figure 2A) to the highest demand well (smaller on the vertical axis). Then, skill switching was accelerated after the well size was kept at the 2nd most demanding well at day 30. The success rate increased over the training. However, it dropped immediately after day 20 and day 30 (Figures 2B,D), where the action selection probability changed, and we refer to this as the recovery valley.

#### 3.1.2. Model comparisons

To examine the contributions of training-induced recovery and spontaneous recovery in Monkey-R's recovery profiles, we evaluated the model predictability based on AIC (Table 1).

**Table 1:** AIC values of each model for Monkey-R.

Model	Training only					
AIC	463.10					
Model	*log (day)	+log (day)	*day	+day	*exp	+exp
AIC	**298.91**	452.21	499.77	514.61	9.808*10^5^	470

AIC of the selected model was shown by the bold text.

The model with the lowest AIC has a logarithmic spontaneous recovery, which influences performance multiplicatively. This model's AIC was considerably different from that of the other model. Thus, we concluded that the contribution of the spontaneous recovery was crucial in Monkey-R's recovery valley.

#### 3.1.3. The estimated performance of Monkey-R

Figure 2E shows the estimated recovery profiles of grip performance, which were latent from the observed behavioral data. Figure 2F shows the predicted success rates over the training days that replicated the recovery and recovery valleys generated from the simulation with the multiplicative logarithmic spontaneous recovery model. The estimated values and the 95% confidence interval (CI) of the parameters are summarized in Table 2.

**Table 2:** The estimated parameter values for Monkey-R.

Parameter	Role	Estimated value	CI
$\alpha_{p}$	Retention rate of the precision grip	0.8466	±0.0110
$\alpha_{c}$	Retention rate of the compensatory grip	0.9135	±0.1018
$\beta_{p}$	Update rate of the precision grip	0.8176	±0.3601
$\beta_{int}$	Interaction term of two skills	0.4085	±0.2198
$\beta_{c}$	Update rate of the compensatory grip	0.5038	±0.2198
$\sigma_{p}$	Motor noise size of the precision grip	0.0158	±0.0110
$\sigma_{c}$	Motor noise size of the compensatory grip	0.0740	±0.0327
$k_{sp}$	Spontaneous recovery	0.2175	±0.0532

For the constant scaling factor of well size, the motor noise size that indicates the accuracy of the precision grip was higher than that of the compensatory grip $\left(\sigma_{p}\ll \sigma_{c}\right)$. Thus, the estimated result reasonably captures the accuracy of the precision grip and the inaccuracy of the compensatory grip.

Because the precision grip training-induced recovery rate $\left(\beta_{p}\right)$ was higher than the compensatory grip training-induced recovery rate $\left(\beta_{c}\right)$, the use-dependent learning ability of the compensatory grip was more impaired than the precision grip. Nevertheless, the motor skill level of the compensatory grip recovered faster than that of the precision grip (Figure 2E). This is because the action selection probability at the initial stage of training was much higher for the compensatory grip than for the precision grip; thus, monkey R had a large chance to train the compensatory grip.

Even though the skill level of the precision grip was lower than that of the compensatory grip, Monkey-R switched from using the compensatory grip to the precision grip after Day 20 (Figure 2C). Thus, switching from behavior with a higher skill level, i.e., the compensatory grip, to other behavior, i.e., the precision grip, caused an initial drop in the success rate (Figures 2D,F). This explains why the recovery valley appeared. Furthermore, the second drop after Day 30 was also explained by an abrupt increase in use of the precision grip after Day 30 (Figures 2D,F).

Because of the positive interaction term between the two skills $\left(\beta_{int}\right)$, the recovery processes for the two skills were complementary such that the preceding recovery of the compensatory grip facilitated the subsequent recovery of the precision grip. Thus, although the opportunity for training the precision grip is very small initially, the skill performance level of the precision grip increased from the outset. Then, when the monkey switched from using the compensatory grip to using the precision grip after the compensatory grip recovered, training-induced plasticity for the precision grip was enhanced by the preceding recovery associated with the compensatory grip. Then, the precision grip skill level converged to that of the compensatory grip (Figure 2E). Thus, Monkey-R succeeded in escaping from the recovery valley in the last stages of training.

There was also a significant contribution of the spontaneous recovery effect on performance $\left(k_{sp}\right)$. Following a certain period after the lesion, precision grip skill was updated by training-induced recovery. In contrast, the spontaneous recovery provided a basis for training-induced recovery to influence executed skill performance (Equation 6), which might also have led to overcoming the recovery valley.

#### 3.1.4. Sensitivity analysis

To examine each model factor's contribution to the recovery valley, we performed a sensitivity analysis regarding how the identified computational model's parameters influenced the recovery profile. Specifically, we examined the effect of the learning rates for the precision and compensatory grips, $\beta_{p}$ and $\beta_{c}$, and the interaction terms of the training-induced performance update, $\beta_{int}$. Additionally, we examined the effect of the retention factor $\alpha_{p},\alpha_{c}$ and the effect of the spontanous recovery $k_{sp}$.

**Figure 3:**
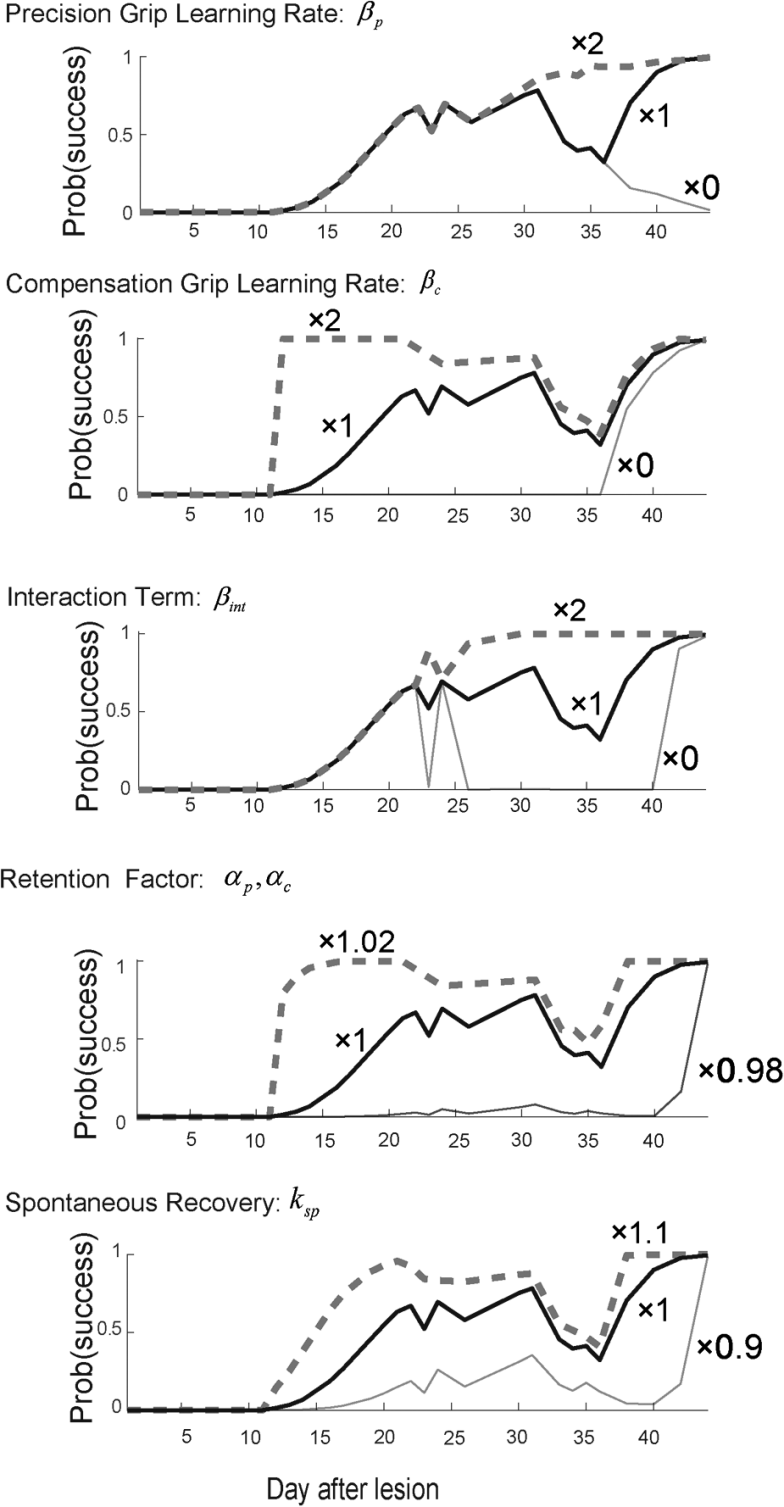
Sensitivity analysis. Top row. Reward probability profiles with perturbations of the precision grip learning rate $\beta_{p}$. The simulation was conducted by changing the best-fitted parameter multiplied by 2, 1, and 0 while other parameters except $\beta_{p}$ remained constant. Second row. Reward probability profiles with perturbations of the compensatory grip learning rate $\beta_{c}$. The simulation was conducted by changing the best-fitted parameter multiplied by 2, 1, and 0 while other parameters except $\beta_{c}$ remained constant. Third row. Reward probability profiles with perturbations of the interaction term $\beta_{\mathrm{int}}$. The simulation was conducted by changing the best-fitted parameter multiplied by 2, 1, and 0 while other parameters except $\beta_{\mathrm{int}}$ remained constant. Forth row. Reward probability profiles with perturbations on the interaction term $\alpha_{p},\alpha_{c}$. The simulation was conducted by changing the best-fitted parameter multiplied by 1.02, 1, and 0.98 while other parameters except $\alpha_{p},\alpha_{c}$ remained constant. Bottom row. Reward probability profiles with perturbation of the spontaneous recovery term $k_{sp}$. The simulation was conducted by changing the best-fitted parameter multiplied by 1.1, 1, and 0.9 while other parameters except $k_{sp}$ remained constant. *Copyright at JNP https://journals.physiology.org/author-info.permissions*.

When the precision grip's learning rate was set twice as large as what it was estimated to be (×2), the second valley of the success probability disappeared, while the first valley around day 25 remained the same (Figure 3, top row). In contrast, when it was set to zero (×0), the success probability decreased drastically after day 30. On the other hand, when the compensatory grip's learning rate was set twice as large as what it was estimated to be (×2), the success probability quickly increased in the initial phase of the training, while it decreased slowly and exhibited the recovery valley (Figure 3, 2nd row). When it was set to zero, the success probability remained at zero until day 35 and then started to gradually increase. Thus, the skill update learning rates for both the precision grip and compensation grip influenced the profile of success probability such that larger learning rates generated faster evolution of the success probability, although the recovery valley was still evident. In contrast, when the interaction term was set twice as large as what it was estimated to be (×2), the recovery valley disappeared for both the 1st and the 2nd drops (Figure 3, 3rd row). Additionally, when it was set to zero (×0), the recovery valley became significantly deeper for both drops. Thus, the interaction term was the most influential in characterizing the recovery valley phenomenon. Meanwhile, the retention factor characterized the speed and asymptotic level of success probability drastically (Figure 3, fourth row). Thus, the retention factor is critical to explain the recovery profile. In addition, the spontaneous recovery influences the success rate (Figure 3, bottom row), while the recovery valley is present even though the contribution was set larger (×1.1) or smaller (×0.9).

### 3.2. Monkey-N

#### 3.2.1. Behaviors

**Figure 4:**
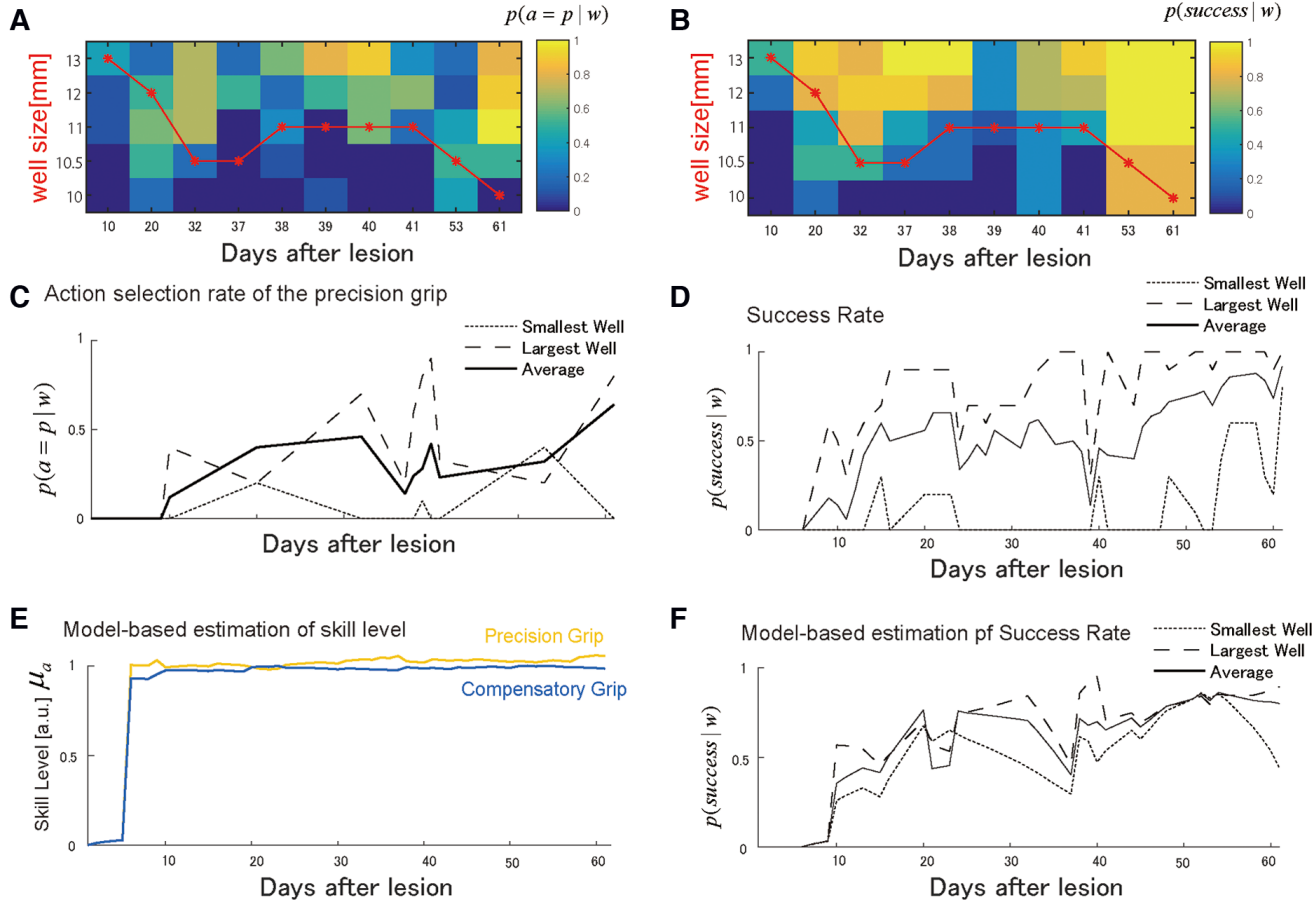
Choice of grasping behaviors for monkey-N. (**A**) Probability of selecting the precision grip in the test sessions over the days for each well size. The red line indicates the size of the wells presented in training sessions. (**B**) The probability of success in the test sessions over days for each well size. (**C**) The average of the action selection probability of precision grip (i.e., action policy) was plotted with it for the smallest and the largest well. (**D**) The average success rate was plotted with it for the smallest and the largest well. (**E**) The estimated development of skill performance $\mu_{c},\mu_{p}$ from the selected computational model. The yellow line denotes the precision grip, and the blue line denotes the compensatory grip. (**F**) The predicted success rate for the largest well (the long dashed), the smallest well (the short dashed), and average sized well (solid). *Copyright at JNP https://journals.physiology.org/author-info.permissions*.

Similar to Monkey-R, Monkey-N (weight: 6.8 kg, dominant hand: left) received a lesion disrupting the grasping representation in M1 of the right hemisphere by injecting ibotenic acid after receiving prelesion training for 13 days. Based on the same analysis conducted with Monkey-R, the probability of selecting either the precision grip or compensatory grip in the test session changed across training sessions such that the monkey initially selected the compensatory grip (Figures 4A,C). After the start of the training, this monkey immediately started using both skills, and the well size largely influenced grip selection probabilities. Interestingly, the backlash after the action selection transition to using the precision grip was observed around day 40 (Figure 4C). At the same time, the success rate drastically dropped (Figure 4D). Subsequently, success rate recovered, which was followed by an increase in the precision grip selection probability.

#### 3.2.2. Model comparisons

We evaluated the model predictability based on AIC (Table 3).

**Table 3:** The estimated parameter values for Monkey-N.

Model	Training only					
AIC	1326.2					
Model	*log	+log	*day	+day	*exp	+exp
AIC	1254.5	**969.6**	1270.6	1270.7	2284.9	1326.2

AIC of the selected model was shown by the bold text.

In terms of the model's predictability, the additive logarithmic spontaneous recovery model was the preferred and selected model. That is, both Monkey-R and Monkey-N had the similar logarithmic function of day for spontaneous recovery contribution while how it influence the motor performance were different.

#### 3.2.3. The estimated performance of Monkey-N

Figure 4E shows the estimated recovery profiles of grip performance using the additive logarithmic spontaneous recovery model. Figure 4F shows the predicted success rates across the training days, which replicated the recovery and recovery valley. The best-fit parameters are summarized in Table 4.

**Table 4:** The estimated parameter values for Monkey-N.

Parameter	Role	Estimated value	CI
$\alpha_{p}$	Retention rate of the precision grip	0.9961	±0.0003
$\alpha_{c}$	Retention rate of the compensatory grip	0.9960	±0.0003
$\beta_{p}$	Update rate of the precision grip	0.0035	±0.0004
$\beta_{int}$	Interaction term of two skills	0.0039	±0.0003
$\beta_{c}$	Update rate of the compensatory grip	0.0037	±0.0004
$\sigma_{p}$	Motor noise size of the precision grip	0.0017	±0.0029
$\sigma_{c}$	Motor noise size of the compensatory grip	0.0184	±0.0141
$k_{sp}$	Spontaneous recovery	0.0684	±0.0447

Like Monkey-R, motor noise magnitude was smaller for the precision grip than for the compensatory grip $\left(\sigma_{p}\ll \sigma_{c}\right)$. There was also a significant contribution of the update rate of the precision grip $\left(\beta_{p}>0\right)$, the compensatory grip $\left(\beta_{c}>0\right)$, and the interaction term $\left(\beta_{\mathrm{int}}>0\right)$, which was also the same as for Monkey-R. The contribution of spontaneous recovery was also significant $\left(k_{sp}>0\right)$.

Skill levels for both grips were large from the initial stage of training, likely due to the additive effect of the spontaneous recovery. This is reasonable since the success probability is relatively high from very initial stage of training (days 6–10) with respect to Monkey R. Nevertheless, since the precision grip's recovery preceded that of the compensatory grip (Figure 4E), switching from the use of both grips on day 32 back to the dominant use of the compensatory grip on day 37 reduced the success rate between days 37 and 39 (Figure 4D,F). Thus, as with monkey R, the recovery valley seemed to be caused by the difference in skill level between two skills and a switch between these skills. The reason why the recovery of the precision grip and the recovery of the compensatory grip (Figure 4E) even though the speeds of the update of the precision grip and the compensatory grip are more or less the same (Table 4) was related to the fact that the interaction term was non-zero (Table 4) and the use of compensatory grip in the early phase (Figure 4C).

#### 3.2.4. Sensitivity analysis

As seen with Monkey-R, the recovery valley with Monkey-N was caused by the difference in the recovery levels between the two skills at the time of the skill switching. This difference in the recovery levels between the two skills should be influenced by the interaction terms. Thus, we performed a sensitivity analysis for each parameter of skill recovery for Monkey-N using the same parameters selected for Monkey-R. Specifically, we examined the effect of the learning rates of precision and compensatory grips, $\beta_{p}$ and $\beta_{c}$, and the interaction term of the training-induced performance update, $\beta_{int}$. Additionally, we examined the effect of the retention factor $\alpha_{p},\alpha_{c}$.

**Figure 5:**
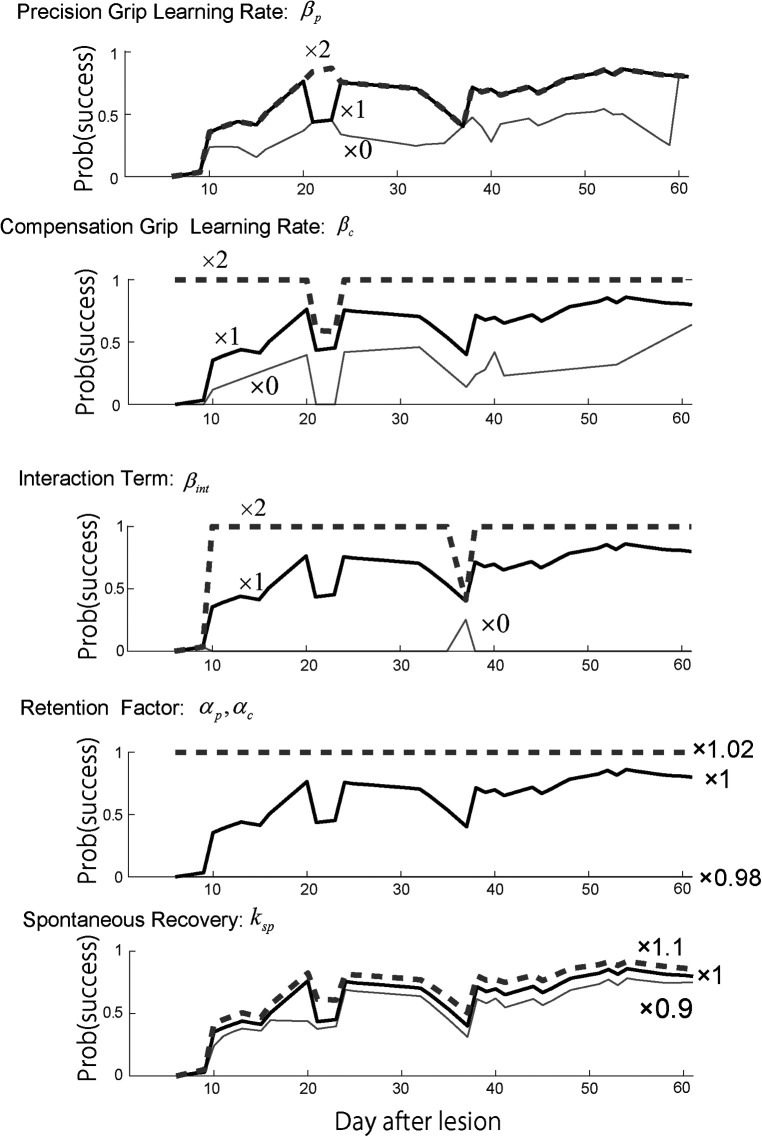
Sensitivity analysis. Top row. Reward probability profiles with perturbations of the precision grip learning rate $\beta_{p}$. The simulation was conducted by changing the best-fitted parameter multiplied by 2, 1, and 0 while other parameters except $\beta_{p}$ remained constant. Second row. Reward probability profiles with perturbations of the compensatory grip learning rate $\beta_{c}$. The simulation was conducted by changing the best-fitted parameter multiplied by 2, 1, and 0 while other parameters except $\beta_{c}$ remained constant. Third row. Reward probability profiles with perturbations of the interaction term $\beta_{\mathrm{int}}$. The simulation was conducted by changing the best-fitted parameter multiplied by 2, 1, and 0 while other parameters except $\beta_{\mathrm{int}}$ remained constant. Forth row. Reward probability profiles with perturbations on the interaction term $\alpha_{p},\alpha_{c}$. The simulation was conducted by changing the best-fitted parameter multiplied by 1.02, 1, and 0.98 while other parameters except $\alpha_{p},\alpha_{c}$ remained constant. Bottom row. Reward probability profiles with perturbation of the spontaneous recovery term $k_{sp}$. The simulation was conducted by changing the best-fitted parameter multiplied by 1.1, 1, and 0.9 while other parameters except $k_{sp}$ remained constant. *Copyright at JNP https://journals.physiology.org/author-info.permissions*.

In contrast to Monkey-R, increasing the precision grip learning rate erased the initial drop of the success rate (Figure 5, top). Then, the increase of the compensation grip learning rate erased the second drop (Figure 5, second) and the increase of the interaction term erased the initial drop (Figure 5, third). Although the contribution of these update rates to each valley was different from those of Monkey R, this sensitivity analysis strongly suggests that these values $\left(\beta_{p},\beta_{c},\beta_{int}\right)$ contributed to the recovery from the drop. In addition, the retention factor was very sensitive to the recovery profile (Figure 5, fourth). While the spontaneous recovery $k_{sp}$ influences the success rate (Figure 5, bottom), while the recovery valley is present even though the contribution was set larger (×1.1) or smaller (×0.9).

According to the fact that the estimated $\alpha_{p},\alpha_{c}$ were large and other parameters were small (Table 4) with respect to these values of Monkey-R (Table 2) and the fact that the result was susceptible to the change of $\alpha_{p},\alpha_{c}$ in the sensitivity analysis (Figure 5), we suggest that Monkey-N's recovery profile was dominated by these retention factors.

## 4. Discussion

To elucidate the computational mechanisms underlying the valley of recovery that has been recognized in clinics (7), we analyzed macaque monkeys' motor recovery of grip skills after stroke. We examined which computational model might reconstruct the changes in reward probabilities. Across two monkeys, both training-induced and spontaneous biological recoveries are necessary to explain the behavioral data. This suggests that the training became effective in improving the performance of grip skills after spontaneous biological recovery was established. This observation is along with the result of skill selection in the previous literature where the untrained monkey (i.e., spontaneous recovery only) exclusively relied on the compensatory grip during the test session (3). Based on the sensitivity analyses of the estimated parameters, both the interaction term and the retention factor were also essential to explain the success probability profile and were influential on the recovery valley profiles. For both monkeys, setting the interaction term at higher levels was effective to avoid the recovery valley. Thus, the dynamics of the memory associated with grip skills and the extent of the generalization of these memories are crucial for overcoming the recovery valley.

In theory, the interaction term facilitates the generalization of learning one skill to performing the other skill. Such a generalization of motor learning has been a central issue in computational motor learning studies (1, 19, 22). Motor learning experiences generalize across postures (33), limbs (34), and movement directions (35). This generalization of learning is mediated by the interaction between two different sensorimotor primitives engaged by executed movements (1, 22). For example, when a certain sensory experience (e.g., skill A) activates corresponding motor primitive neurons, a different sensory experience (e.g., skill B) may also partially activate the same primitives because the representation of two primitives overlap. As a result, an experience of B leads to a partial memory update of A, and then the learning of B generalizes to the learning of A (1, 21, 22). Such overlapping representations of motor primitives appear in the population coding of motor commands in the motor cortex (36) where the gains of neural activities were tuned as a function of the movement direction of the hand: the experience of direction A partially activated the neurons for direction B. A similar population coding was found for finger and wrist movements. For instance, the neurons in the motor cortex with a preferred direction on the thumb finger were partially activated by index finger movements (23). Thus, when a learning experience of the index finger was accumulated *via* compensatory grip training, this training effect should generalize to the coordinated movements of the thumb and index fingers for the precision grip movement since these two skill primitives were likely to overlap. Accordingly, the generalization abilities of the two grip skills are determined by the condition of the motor representations, such as the overlap in the two skills in the peri-infarct cortex or the amount of intact neural resources related to these skills. Such different conditions in the motor cortex may have caused two different generalization abilities in the two monkeys that we analyzed in this paper. Notably, the neuron groups associated with movement (index finger and thumb fingers) areas active for the two grip tasks can dramatically change across tasks, since these skill switches change the contextual input to the motor systems. Thus, some neurons are only active for one of the two skills, which was illustrated in a drastic change in M1 neuron activity between two contexts: posture vs. reaching (37). This partial overlap may explain partial learning of the two motor skills (38). Such a partial transfer of the learning skills between the index and thumb fingers may be more than simple interaction term since it actually generates very complex profiles of the valley and recoveries and slightly different profiles between the two monkeys. Note here that our modeling relies on the conventional theory of M1 function for representing motor commands (39). The modern theory of the function of M1 focuses on the likely contribution of the dynamical aspect (40) to the feedback control loop (41, 42). Thus, the latent variables of our model $\mu_{\left[p,c\right]}$ could be the performance level of the motor policy rather than the feedforward component of the executed movements. Since we focused on the recovery level of skill representation, we excluded the effects of optimization and strategy, which are also important for performance of the skill (20). These limitations of our modeling may have caused the estimation error in the recovery profiles (Figures 2F,4F). However, since both monkeys received a lesion focally in the motor cortex, considering only the skill execution, excluding the optimization and strategy considered in the higher motor area or prefrontal area seems reasonable. At least, the bottom-line conclusion from this model-based analysis is that dynamics of the interaction between two motor skills represented in the motor cortex is crucial for generating the recovery valley.

The restoration of blood flow and resolution of edema within the penumbra, i.e., the area of reversibly damaged tissue surrounding the irreversibly damaged core, is thought to underlie spontaneous recovery after stroke (43). In the monkeys whose data were reanalyzed in the present study, however, these changes in the penumbra were not the primary cause of spontaneous recovery because the neurons in the motor cortex had been directly destroyed by ibotenic acid without affecting blood flow. Therefore, plastic changes in neuronal structure and function were probably involved in spontaneous and training-induced recovery in the monkeys in the present study. The expression of growth-associated genes, which is important in structural changes of neurons, is known to increase in the region surrounding brain damage (44) and is suggested to be involved in spontaneous recovery. Upregulated gene expression was mainly observed several weeks after brain damage and therefore is thought to be involved in plasticity during the acute phase of brain damage. However, most of the previous studies were performed in rodent models of brain damage, and further investigations are needed to understand the time course of gene expression changes after damage in the primate brain. Moreover, some of the growth-associated genes are constantly expressed in the normal adult brain. For example, the expression of growth-associated protein-43 (GAP-43), whose expression has been correlated with axonal sprouting (45), is most abundant during the developmental period, but a certain level of expression has been found in monkey neocortical areas and the hippocampus (46–48). The constant expression of growth-associated genes may underlie spontaneous recovery after the acute phase.

Although our computational model of motor recovery explained the recovery dynamics of motor skills, we found that the most critical problem for causing the recovery valley was anti-optimal decision-making from the compensatory grip to the precision grip (Figures 2C,E). This skill selection mechanism between the grips during training and the reason why it was anti-optimal still remain unclear. The precision grip, which uses the pad of the thumb and the pad/side of the index finger, is the most frequently used grip type when monkeys manipulate small objects, whereas the compensatory grip that we observed in the early phase of recovery is not commonly used in healthy macaque monkeys (49, 50). Thus, the preference for the use of the precision grip after recovery is reasonable. Particularly, due to the interaction term of the skill development, after the compensation grip training facilitated the precision grip's recovery, the use of the precision grip resulted in successful food retrieval, which increased motivation for using the precision grip. If the interaction term is not influential, skill switching results in a drop in the success rate, which discourages the sustainable use of the precision grip. This switching scenario between precision and compensatory grips is analogous to learned nonuse, where stroke patients exhibit excessive reliance on the unaffected limb compared to the paralyzed limb after stroke (51). A computational model of learned nonuse has been theorized with a reinforcement learning model (52). In this model, the value representation of each skill is updated to maximize the expectation of value by a reinforcement learning algorithm. In fact, arm choice in humans is determined by the learned values and efforts (53). Additionally, in stroke patients, the performance of the limb measured immediately after therapy may predict the long-term improvement in arm use (54). Based on these results of learned nonuse, the macaque's selection of two grip skills that we examined in the present paper might also be mediated by the value-based reinforcement learning algorithm. However, to examine this idea, a further experiment where we manipulate the quantity and quality of rewards and examine decision-making responses with reward changes is necessary.

Our modeling study and simulations may suggest several clinical applications. Based on our sensitivity analyses, increasing interaction terms may avoid the drop in performance during recovery. Thus, clinical interventions that increase the interaction term between two skills and between two limbs is expected to prevent the recovery valley and achieve efficient rehabilitation for precision. As discussed above, the generalization effect of motor learning is generated by overlaps in the neural representations of two motor primitives. Thus, somehow extending the overlap of representations of the two skills or virtually replicating such a wider overlap should enhance the interaction effects. According to motor adaptation theory, generalization patterns are characterized by the profile of the tuning function of the receptive fields (22). For instance, applying transcranial direct current stimulation (tDCS) to the cortical motor cortex increases potentiation of a synapse (55) by reducing γ-aminobutyric acid (GABA) concentrations (56), which may modulate the reciprocal connections of neurons that are crucial for motor learning (57). Thus, tDCS on the motor area may enhance interaction terms between the ideal motor skill and the compensatory motor skill. Indeed, applying tDCS on the motor cortex increases generalization across motor learning movements (58). Another possibility for realizing such an expansion of the overlap between two motor primitives is that when the compensatory motor skill is selected in the early phase of the rehabilitation, the primitive for the ideal motor skill could be stimulated along with the movement of the compensatory motor skill. For instance, when the compensatory grip is selected where only the index finger's motion is initiated, if the thumb is moved compulsorily by an assistive robot for the grip movement such as (59), the proprioceptive input to the sensorimotor primitive of the thumb forms a sensory experience of the precision grip resulting in a corresponding stimulation of the precision grip primitive. This would replicate the interaction between the two grip skills and may lead to a generalization of skill learning from the compensatory grip to the precision grip.

In addition, the present study suggests that enhancing the retention factor will be a key strategy to increase the effects of training and to prevent the recovery valley. Previous studies have demonstrated that activity-dependent neural plasticity, such as LTP, is essential for the long-term retention of learned motor skills (60, 61); therefore, interventions to upregulate activity-induced plasticity are thought to enhance this parameter. A line of studies has suggested that neuromodulation techniques such as transcranial magnetic stimulation (TMS) and tDCS increase the magnitude of LTP (62). A recent study reported that the collapsin response mediator protein 2 (CRMP2)-binding compound edonerpic maleate facilitated experience-driven synaptic glutamate α-amino-3-hydroxy-5-methyl-4-isoxazole-propionic-acid (AMPA) receptor delivery and accelerated training-induced motor function recovery from brain damage in mice and monkeys (63). These reports suggest the clinical validity of neuromodulation- and medication-mediated interventions to accelerate the effects of rehabilitative training. An important next step will be to explore how these intervention technologies affect this parameter. The information will be clinically useful in estimating the effect size of the interventions on each patient's outcome.

There are some limitations in our modeling study. First, since the days that we had video recording data were limited, we analyzed the action selection only for these data. Thus, we assumed that the action selection probability between these days was constant. We considered this assumption reasonable since the action selection probability shifted gradually over the training periods (Figures 2,4C). Second, since the two monkeys' lesion areas are slightly different, the two monkeys exhibited very different recovery profiles (Figures 2,4C,D). Nevertheless, our model-based analysis derived a common basis between these two monkeys: the contributions of both spontaneous and training-induced recoveries and the importance of the interaction between the two skills. Whereas there was randomness in the recovery profile of the Monkey-N (Figure 4D), the computational model filtered such randomness out and captured the recovery valley (Figure 4F). Such a large lesion in Monkey-N made the difference in the recovery and the choice profiles between the two grip skills unclear: Monkey-N selected the precision grip from the initial stage of the training exhibiting a certain success rate. Thus, our model estimated the skill level between the two grips to be more or less the same. Third, in our modeling, we focused only on skill recovery when the choice of skills was changed during the training without modeling the decision-making process. For instance, although, in Monkey-R, the recovery rate of the precision grip is higher than that of the compensatory grip, and thus it might be reasonable to select the precision grip to increase its performance, this monkey-R initially selected the compensatory grip. However, our modeling does not answer for the elucidation of this process. Since this decision-making process is important for understating the rehabilitation process, we would like to continue to study this problem for our future work. Fourth, the model was examined only for the monkey model of the stroke where the lesion was made focally in the hand area in the primary motor cortex. Thus, this model does not capture heterogeneous phenomena that happen in human stroke recovery; thus, the generalization of our conclusion has a significant limitation. Fifth, the estimated contribution of spontaneous recoveries was very different between the two monkeys: Whereas Monkey-R's spontaneous recovery was estimated as contributing multiplicatively, Monkey-N's was estimated as additively. This difference leads to very different profiles of the recovery of skill levels. More importantly, the interpretation of the effect of spontaneous recovery is very different. We speculate that there are two different roles in spontaneous recovery: One is a spontaneous biological recovery where the spontaneous reorganization of neural systems forms a basis to re-learn representations of the skills, which thus contributes multiplicatively, and the other is a spontaneous skill learning where the previous experience of the skill was formed in an off-line manner which was added onto the use-dependent skill recovery. Since Monkey-N's lesion was large, the spontaneous recovery for forming the basis of the representation might not be successful. Sixth, we assumed that the motor noise values were constant thought the training days. Although it is possible that the motor noise magnitude changed over training, our system modeling approach does not allow changing these values while estimating the hidden representation of skills. Thus, this is a significant limitation of our study.

## 6. Conclusion

The present study explored computational mechanisms underlying a transient drop in task performance and the emergence of compensatory grips that differed from those used before lesion induction that was observed in macaque monkeys (3), which have also been shown in the squirrel monkey model of primary motor cortex lesions (5, 6). We found that spontaneous recovery, training-induced recovery, retention factors, and interaction terms are crucial to explaining profiles of recovery and recovery valleys through model selection, parameter estimation, and sensitivity analyses. Furthermore, the simulation-based examination of the model parameters provided clues for effective rehabilitation techniques, including effective medications, brain stimulation, and robotic rehabilitation technologies.

## Data Availability

The data used for the analysis are available from the corresponding author upon reasonable request.
